# Identification and pathogenicity analysis of a novel intronic *COL4A5* variant in a Chinese family

**DOI:** 10.3389/fmed.2026.1783004

**Published:** 2026-05-07

**Authors:** Pei Qian, Hui-mei Huang, Lei Suo, Zhijuan Li, Min Zhang, Ying Bao

**Affiliations:** Department of Nephrology, Xi’an Children’s Hospital, Affiliated Children's Hospital of Xi'an Jiaotong University, Xi'an, China

**Keywords:** aberrant splicing, Alport syndrome, *COL4A5*, minigene, RT-PCR

## Abstract

**Background:**

X-linked Alport syndrome (XLAS) is a disorder of type IV collagen structure caused by pathogenic variants of the COL4A5 gene and characterized by progressive kidney disease, hearing loss, and ocular abnormalities. Although mutation screening is commonly performed for AS-associated genes, transcriptional analysis is not a standard test for patients with XLAS, and the functional consequences of splicing abnormalities caused by intronic variants are rarely studied.

**Methods:**

In this study, nine family members from a three-generation pedigree in Gansu Province, China, were investigated. We used targeted next-generation sequencing to identify genetic variants in family members, confirmed the mutation site by Sanger sequencing, and predicted the pathogenicity of the variant using bioinformatics software. The splicing effects of the mutation were analyzed using minigene testing with HEK 293T cells, and further confirmed by *in vivo* transcript analysis using RNA extracted from the proband’s skin cells.

**Results:**

The proband was a 5-year-old male child who presented with microhematuria at the age of 4.5 years. Four relatives in this pedigree had a family history of kidney disease, all presenting with microhematuria with or without proteinuria. The proband’s elder brother and maternal uncle had progressed to kidney failure. Immunofluorescence staining of the proband’s epidermal basement membrane showed negative expression of the α5 chain. A novel splice variant (c.1587+4A>G) was identified in intron 23 of COL4A5 among the affected family members. Sanger sequencing confirmed that this variant co-segregated with the disease phenotype. In silico analysis predicted that this variant may cause abnormal splicing. Transcript analysis of the proband’s skin tissue and minigene assay demonstrated that the variant causes abnormal mRNA splicing and exon 23 skipping, leading to a frameshift, premature termination codon, and predicted nonsense-mediated mRNA decay.

**Discussion:**

The study identified a novel intronic variant (c.1587+4A>G) in the COL4A5 gene in a Chinese family with XLAS, and functional experiments verified that this variant induces aberrant splicing. This study highlights the importance of transcript analysis for intronic variants and further expands the mutational spectrum of COL4A5 in XLAS.

## 1 Introduction

Alport syndrome (AS) is an inherited type IV collagen disorder characterized by hematuria, proteinuria, and progressive kidney failure, accompanied by sensorineural hearing loss (SNHL) and specific ocular abnormalities in some patients (1). Pathogenic variants in the *COL4A5* gene (NM_033380.3), which encodes the type IV collagen a5 chain, cause X-linked Alport syndrome (XLAS)—traditionally regarded as the most common form of AS (2). However, a study based on the Genome Aggregation Database (gnomAD) of more than 122,000 subjects showed the global prevalence of autosomal dominant (AD) AS at 1 in 106 and that of the X-linked form at 1 in 2,320 (3). Recent research also indicates that AD Alport syndrome is highly prevalent in Singapore’s multiethnic population, at 1 in 165 overall and the highest in the Chinese subgroup (0.808%, ~1 in 124) (4). The clinical diagnosis of AS is mainly based on clinical manifestations and kidney histopathology. Owing to the invasiveness of kidney biopsy, its application is limited. In contrast, genetic testing has become the preferred diagnostic strategy because it has higher sensitivity and specificity for AS diagnosis and can provide predictive information about disease severity and prognosis. In males with XLAS, nonsense variants are associated with the most severe early-onset end-stage kidney disease (ESKD) phenotype, splice-site variants with an intermediate phenotype, and missense variants with the mildest phenotype, with median ages at kidney failure of 18, 28, and 40 years, respectively (5, 6). In addition, there are significant differences in kidney survival based on the abnormal splicing pattern of the transcript (6, 7). Some retrospective studies have reported that the proportion of patients with splicing variants in XLAS can be as high as 13.7–18.2% (5, 8, 9). These findings emphasize the importance of identifying splicing variants of *COL4A5*. Few reports have focused on the function of intronic variants, making it difficult to distinguish intronic variants that cause splicing errors from harmless polymorphisms. The former variants are often classified as variants of uncertain significance (VUS), leading to missed diagnoses and underestimation of the clinical phenotypes of some patients. In this study, we identified an intronic variant of the *COL4A5* gene in a Chinese family using whole-exome sequencing (WES). We adopted a dual strategy combining *in vivo* transcript analysis and *in vitro* splicing assay to confirm the potential induction of abnormal splicing by this variant, thus providing functional evidence for its pathogenicity.

## 2 Materials and methods

### 2.1 Clinical data

**Figure 1:**
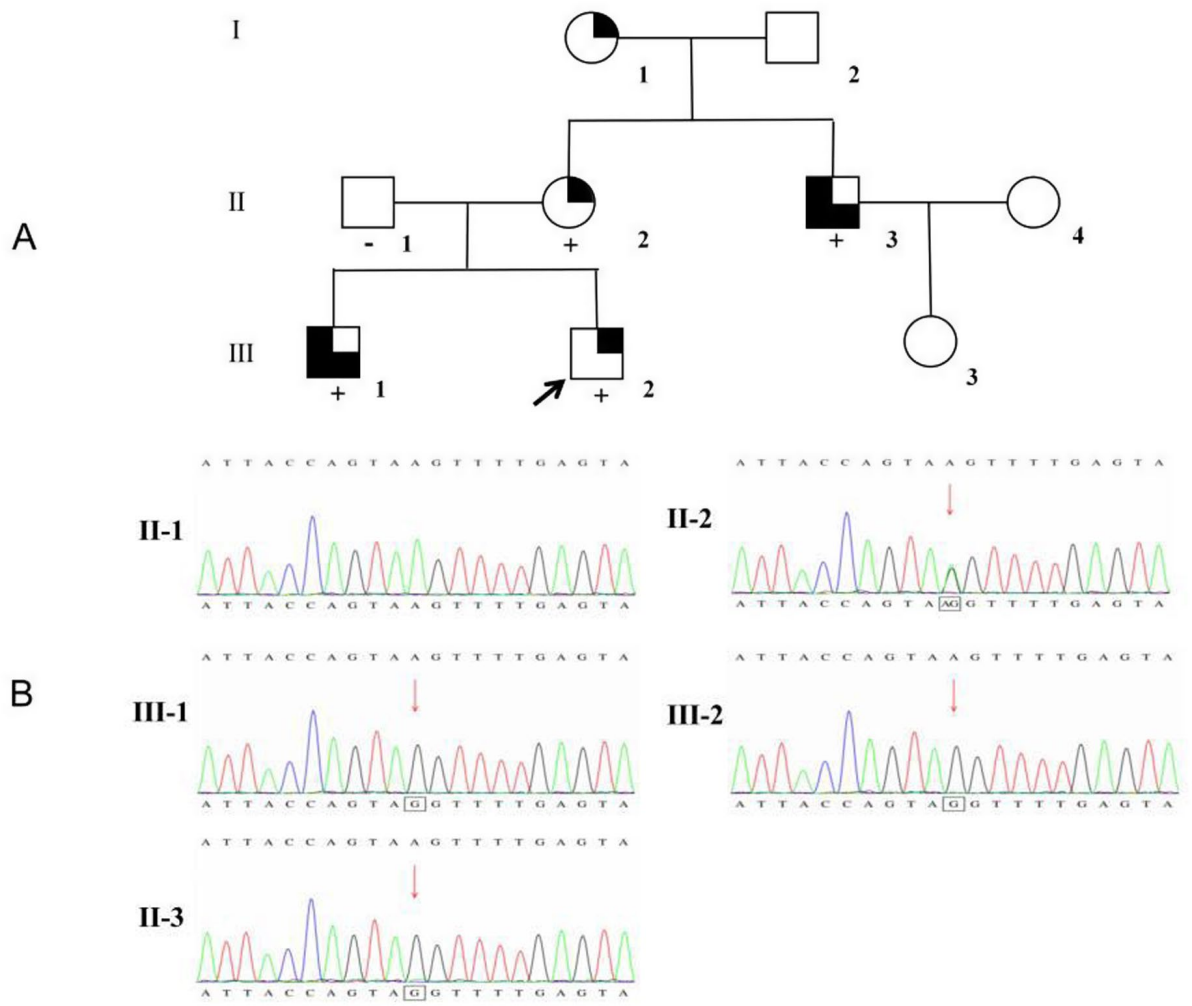
Pedigree of the proband’s family and identification of the *COL4A5* gene variant. **(A)** Pedigree analysis of the family. The upper-right black quadrant indicates microhematuria, the lower-right black quadrant indicates microhematuria combined with proteinuria, the upper-left quadrant indicates kidney failure, and the lower-left quadrant indicates sensorineural hearing loss. White symbols represent individuals with a normal phenotype. Symbols “−” and “+” denote wild-type and variant-carrying individuals, respectively. The proband is indicated by a black arrow. **(B)** Sequencing results showing the *COL4A5* mutations in the proband and his family members.

This study investigated a three-generation pedigree of a Chinese family, consisting of nine relatives (Figure 1A). We collected clinical data from all participants, including sex, age at onset, kidney function parameters (hematuria, proteinuria, and chronic kidney disease stage), sensorineural hearing loss, and ocular lesions. The staging of chronic kidney disease stage is defined by the KDIGO clinical practice guidelines based on glomerular filtration rate (GFR) and evidence of kidney damage (10). GFR was calculated using the original Schwartz formula (11).

### 2.2 Whole-exome sequencing and variant confirmation

WES was performed on a blood sample from the four affected individuals (II-2, II-3, III-1, III-2) and one healthy subject (II-1). Initially, DNA was fragmented to create a sequencing library. Whole-exome capture was performed using the IDT xGen Exome Research Panel v1.0. Sequencing was carried out on an Illumina NovaSeq 6,000 platform with paired-end 150 bp (PE150) reads. The sequencing coverage of the target region was ≥99%. Stringent quality control standards were applied to the sequencing data, requiring an average sequencing depth of 200 × for the targeted regions, with at least 98.5% of the sites having an average depth exceeding 20×. Sequencing reads were aligned to the UCSC hg19 human reference genome using the Burrows-Wheeler Aligner software, followed by removal of PCR duplicates. Base quality score recalibration and variant calling were subsequently conducted using the Genome Analysis Toolkit (GATK), enabling the identification and genotyping of single nucleotide variants and insertion–deletion polymorphisms (INDELs). The identified variants were further annotated bioinformatically using multiple public mutation and population frequency databases, including ClinVar, HGMD, gnomAD, the Exome Sequencing Project, and the 1,000 Genomes Project. The potential pathogenic or deleterious effects of these variants were predicted based on integrated information from these databases. Variants with potential pathogenic significance were screened, and classified in accordance with the guidelines for sequence variant interpretation co-published by the American College of Medical Genetics and Genomics and the Association for Molecular Pathology, with additional refinement based on consensus recommendations from the ClinGen Sequence Variant Interpretation Working Group and the Association for Clinical Genomic Science. All detected variants were subsequently validated by Sanger sequencing to confirm their authenticity.

### 2.3 In silico splicing assay

We used publicly available bioinformatics software analysis tools to evaluate the potential impact of the identified variant on pre mRNA splicing. These tools include the SpliceAI^1^, RDDC^2^, BDGP^3^, and NetGene2.^4^

### 2.4 *In vitro* minigene assay

A series of minigene plasmids harboring the *COL4A5* variant (NM_033380.3: c.1587 + 4A > G) was designed and constructed. The inserted fragment spanned exon 22 (93 bp), intron 22 (1,396 bp), exon 23 (71 bp), intron 23 (308 bp), and exon 24 (192 bp). The wild-type minigene fragment was PCR-amplified from human genomic DNA (gDNA) and cloned into the BamHI/XhoI double-digested pMini CopGFP vector. Mutant constructs were generated via site-directed mutagenesis. All plasmids were validated by Sanger sequencing with fully correct sequences confirmed. Primer sequences are shown in Table 1. The red-highlighted sequence represents the gene-specific region of the *COL4A5*-WT/F/R primers that anneals to the target genomic DNA. The minigene plasmids were transfected into 293 T cells. After 6 h, the medium was replaced with fresh complete medium. Total RNA was extracted 48 h later, reverse-transcribed into cDNA, and the target fragment was amplified by RT PCR. Products were analyzed via agarose gel electrophoresis and Sanger sequencing to determine the effect of the variant on *COL4A5* pre mRNA splicing.

**Table 1:** The primer sequences used in the minigene assay.

Primer name	Sequence (5′→3′)
*COL4A5*-WT-F	AAGCTTGGTACCGAGCTCGGATCCGTGACAAAGGTGACACTTGCTTCAACTGCATTGGA
*COL4A5*-WT-R	TTAAACGGGCCCTCTAGACTCGAGAGGCTCTCCTTTCGGGCCAGGAAGCCCTGGCAAT
*COL4A5*-MT-F	TACCAGTAgGTTTTGAGTATATTATAAAACAAAAAGAAGTAGA
*COL4A5*-MT-R	CTCAAAACcTACTGGTAATCCTTTGGGACCAG
Mini*COL4A5*-RT-F	GGCTAACTAGAGAACCCACTGCTTA
Mini*COL4A5*-RT-R	AGGCTCTCCTTTCGGGCCAG

### 2.5 RT-PCR analysis

Total RNA was extracted from the proband’s skin tissue and peripheral blood of a healthy control using TRIzol reagent (Invitrogen, Carlsbad, CA, United States) in accordance with the manufacturer’s protocol. First-strand cDNA was synthesized from 1 μg of total RNA using the PrimeScript RT Reagent Kit (Takara, Dalian, China) with random hexamer primers in a 20 μL reaction system. RT-PCR was performed to amplify the region encompassing the *COL4A5* c.1587 + 4A > G variant (NM_033380.3) using specific primers (forward: 5′-TCCTGGAGAAAGGGGTCAGA-3′; reverse: 5′-TGTCCAGGAGTGCCAGGTAA-3′). PCR amplification was carried out in a total volume of 25 μL containing cDNA template, primers, and PCR master mix under the following cycling conditions: initial denaturation at 95 °C for 3 min; 35 cycles of 95 °C for 30 s, 58 °C for 30 s, and 72 °C for 45 s; followed by a final extension at 72 °C for 5 min. PCR products were separated by 2% agarose gel electrophoresis and visualized under UV illumination. Bands of interest were purified and subjected to direct Sanger sequencing to assess transcript alterations and potential aberrant splicing events.

### 2.6 Immunofluorescence staining of type IV α5 collagen in the epidermal basement membrane

Immunofluorescence staining was performed on frozen sections of the proband’s skin tissue: 3-μm-thick frozen sections were immunostained using FITC-labeled mouse anti-human type IV collagen α5 chain monoclonal antibody (Cat. No. ab231957, Abcam, United States) and FITC-labeled mouse anti-human type IV collagen α1 chain monoclonal antibody (Cat. No. ab231957, Abcam, United States). The procedure was performed as previously described (12).

## 3 Results

### 3.1 Clinical features and pedigree analysis

**Figure 2:**
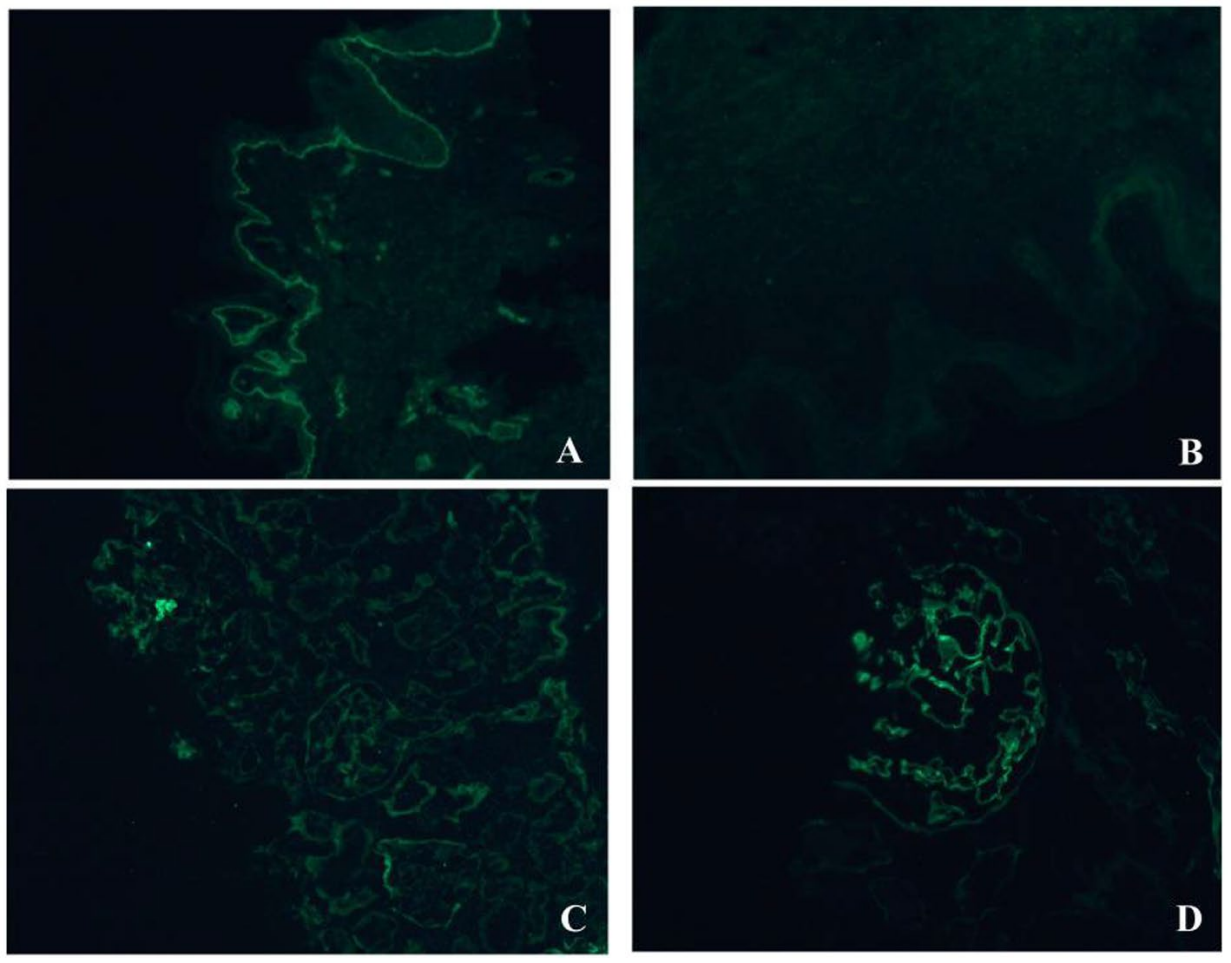
Immunofluorescence staining of type IV collagen α1 and α5 chains in skin and *kidney* basement membranes. **(A)** Positive immunofluorescence staining of type IV collagen α1 chain in the proband’s skin basement membrane (green fluorescence, linear distribution along the basement membrane). **(B)** Negative immunofluorescence staining of type IV collagen α5 chain in the proband’s skin basement membrane (no detectable green fluorescence signal). **(C)** Positive immunofluorescence staining of type IV collagen α1 chain in control *kidney* tissue (green fluorescence, linear/granular distribution along the glomerular and tubular basement membranes). **(D)** Positive immunofluorescence staining of type IV collagen α5 chain in control *kidney* tissue (green fluorescence, linear/granular distribution along the glomerular and tubular basement membranes). Note: α1 immunofluorescence staining (×200); α5 immunofluorescence staining (×200).

The family pedigree is shown in Figure 1A. The proband, Individual III-2 (aged 5 years), was admitted to Xi’an Children’s Hospital with persistent microscopic hematuria. Microscopic hematuria was discovered at age 4.5 years. The proband’s urine contained 200–300 red blood cells/μl. Urine morphology revealed 30–40 cells/HP with 100% dysmorphism, but no proteinuria was present. Complete blood count, biochemistry, or immunology tests were normal. Pure tone audiometry and ophthalmological examinations were normal. No obvious abnormalities were observed in abdominal ultrasonography, color Doppler vascular ultrasonography, or electrocardiography. Urinalysis revealed microscopic hematuria in the proband’s mother (II-2) and maternal grandmother (I-1). The proband’s older brother (III-1) developed microscopic hematuria at the age of 4 years and kidney failure at 15 years old. The proband’s uncle (II-3) developed kidney failure at 25 years old. The proband’s father (II-1) and maternal grandfather (I-2) had no signs of kidney disease. Kidney function was normal in all but the proband’s older brother and maternal uncle. Furthermore, the proband’s maternal uncle (II-3) and older brother (III-1) had high-frequency SNHL (Figure 1A; Table 2). Given that the proband had microscopic hematuria, five family members had a family history of kidney disease, and two relatives in the family had a history of kidney failure, XLAS was suspected. As the proband refused kidney biopsy, immunofluorescence staining of type IV collagen in skin tissue was performed instead. Immunofluorescence staining of the proband’s skin showed positive α1(IV) expression, confirming intact basement membrane structure and reliable staining results. Due to ethical and invasive limitations, fresh healthy skin controls were unavailable. We instead used *kidney* tissues from pediatric patients with nephrotic syndrome as alternative controls, stained with the same mouse anti-human α1(IV) and α5(IV) monoclonal antibodies. Clear positive staining for both chains in control *kidney* tissue verified the high specificity and binding activity of the antibodies. These results exclude false-negative signals caused by antibody dysfunction, supporting the reliability of our findings. The absent α5(IV) staining in the proband’s skin basement membrane thus indicates a genuine α5 chain defect rather than antibody abnormality (Figure 2).

**Table 2:** Clinical features of affected individuals in the family.

Pedigree	Sex	Ocular examination	Audiometry	Manifestation of kidneys
I-1	Female	Normal	Normal	Microhematuria
II-2	Female	Normal	Normal	Microhematuria
II-3	Male	Normal	SNHL	Microhematuria, proteinuria Progressed to CKD 5 by age 25
III-1	Male	Normal	SNHL	Microhematuria, proteinuria Progressed to CKD 5 by age 15
III-2	Male	Normal	Normal	Microhematuria

CKD, chronic kidney disease; SNHL, sensorineural hearing loss.

### 3.2 Mutation analysis

WES results revealed a novel hemizygous variant (chrX:10784 0302, NM_033380.3: c.1587 + 4A > G) in intron 23 of the *COL4A5* gene in the proband. Further pedigree verification by Sanger sequencing revealed that the proband’s older brother and maternal uncle were both hemizygous for the variant. This variant was not found in the proband’s unaffected father, and the proband’s mother was heterozygous for the mutation. The phenotype and genotype of the proband and three patients cosegregated within the family (Figure 1B). This variant (c.1587 + 4A > G)—an intronic variant not previously reported to be associated with any diseases—was absent from the public databases including dbSNP, ExAC and the 1,000 Genomes Project, and is thus classified as a rare variant. Furthermore, according to the standards and guidelines of the American College of Medical Genetics and Genomics, this variant was categorized as a VUS (PM2-Supporting+PS1-Supporting+PP1-Moderate).

### 3.3 In silico splicing analysis

To assess the potential effect of c.1587 + 4A > G on mRNA splicing, SpliceAI analysis was performed, which predicted a donor loss score of 0.6, indicating that the variant is highly likely to disrupt the canonical splice donor site. The RNA splicing prediction tool, developed by China’s Rare Diseases Data Center (RDDC), indicated that this variant could disrupt the splice donor site, resulting in exon 23 skipping. This alteration ultimately triggers a frameshift mutation, leading to premature translation termination. The online BDGP software predicted that the original splice site was lost after the mutation, suggesting a potential impact on RNA splicing. Additionally, the online NetGene software showed a change in the splice site score before and after the mutation, which also implied a possible effect on splicing.

### 3.4 *In vitro* splicing analysis

**Figure 3:**
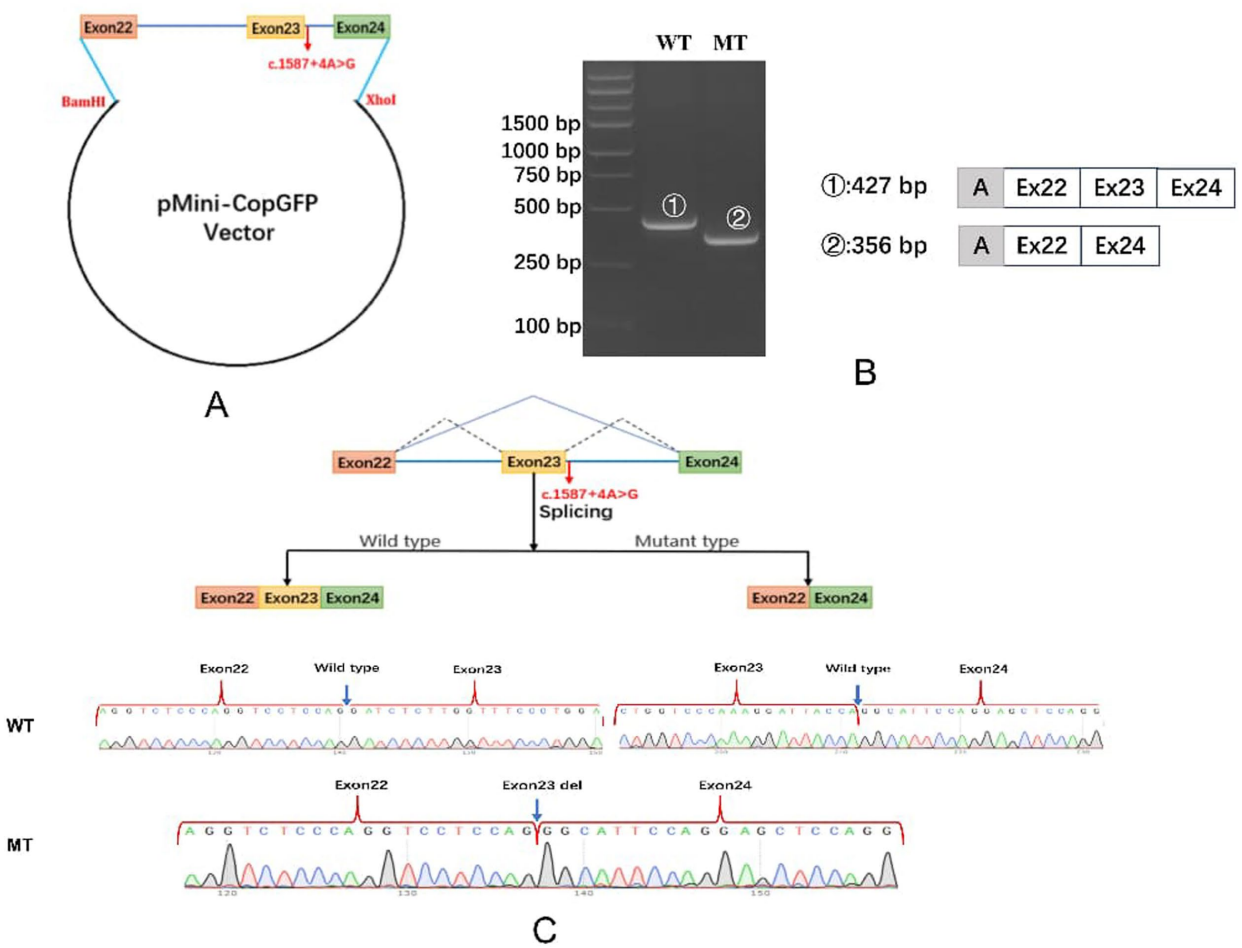
Minigene-assay transcript analysis. **(A)** *COL4A5* exons 22–24, spanning the intron variants, were cloned into the pMini-CopGFP vector. **(B)** Agarose gel (2%) electrophoresis of the reverse transcription-polymerase chain reaction (RT-PCR)products obtained from RNA of HEK 293 cells transfected with the wild-type (WT) or mutant-type (MT) minigene vector. WT exhibited a single full-length band, and MT exhibited a single band (exon 23 skipping). The drawing on the right shows the predicted WT and MT amplicon sizes. **(C)** Schematic diagram showing the variation and its consequences: Upper panels show schemas of aberrant splicing (blue lines). Normal splicing is indicated by black dotted lines. Sequencing chromatogram of *COL4A5* wild-type cDNA and splice variants are shown in the lower panels. Sequencing traces of the 427 bp fragment (WT) with normal splicing and the 356 bp fragment (MT) in which exon 23 is skipped. The variant (c.1587 + 4A > G) disrupted the splice donor site of intron 23, resulting in exon 23 skipping, which creates a transcript with a 71-bp deletion.

We amplified a DNA fragment spanning exons 22–24 of *COL4A5* and cloned it into the minigene pMini-CopGFP vector (Figure 3A). Following transfection, the WT and MT (c.1587 + 4A > G) splice region variants were functionally evaluated in cultured cells (see Materials and Methods). Agarose gel electrophoresis (Figure 3B) showed that, following splicing of the intronic sequence, cells transfected with the WT vector produced the expected 427-bp amplicon encompassing the vector’s endogenous exons and the intervening *COL4A5* exon 22–24 sequences. In contrast, cells transfected with the MT vector produced a 356-bp amplicon encompassing the vector’s endogenous exons and the intervening *COL4A5* exon 22 and 24 sequences, with the entire 71-bp sequence of exon 23 deleted (Figure 3C). The variant (c.1587 + 4A > G) destroyed the splicing donor site of intron 23, resulting in skipping of exon 23.

### 3.5 *In vivo* RT-PCR analysis

**Figure 4:**
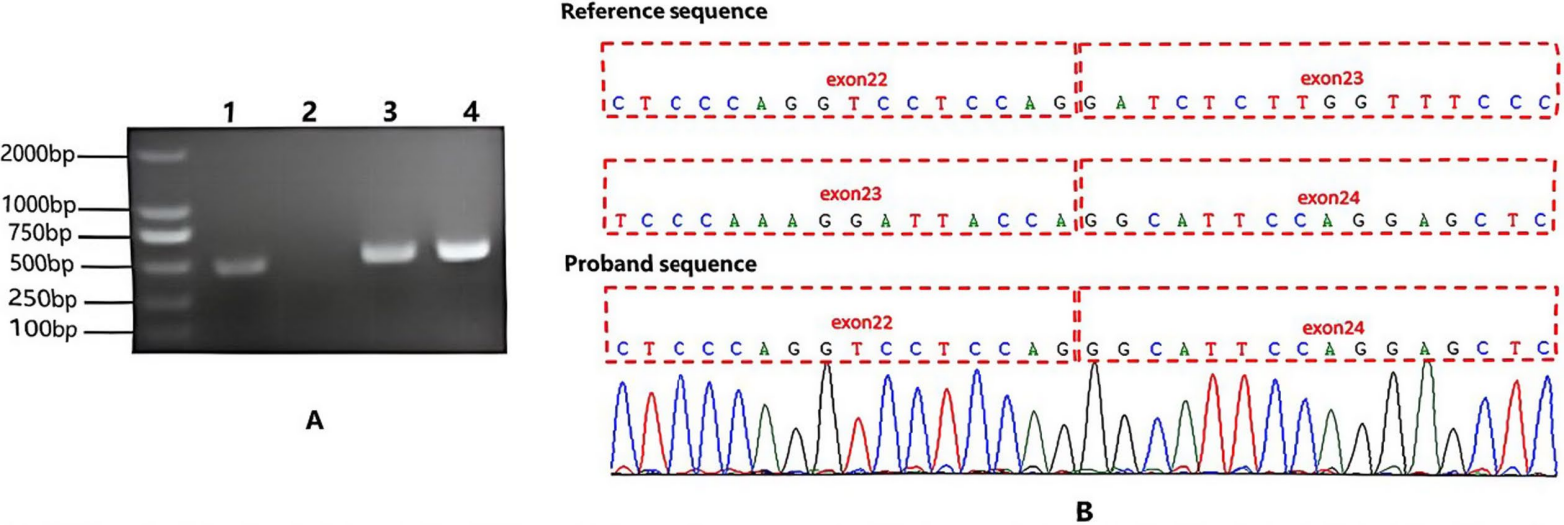
RT-PCR and Sanger sequencing analysis of *COL4A5* transcripts in the proband’s skin tissue. **(A)** Agarose gel electrophoresis of RT-PCR products. The expected fragment sizes were 537 bp for the target gene *COL4A5* (amplified in lanes 1 and 2) and 508 bp for the reference gene *WDR45* (amplified in lanes 3 and 4). Lane 1: Proband’s skin tissue (positive *COL4A5* band migrating faster than both the expected wild-type transcript and the *WDR45* internal control), confirming *COL4A5* mRNA expression and indicating an aberrant splice variant. Lane 2: Healthy control’s peripheral blood (no visible *COL4A5* band), verifying the absence of *COL4A5* expression in blood cells. Lane 3: Proband’s skin tissue (positive WDR45 band, ~508 bp), validating RNA integrity and RT-PCR efficiency. Lane 4: Healthy control’s peripheral blood (positive *WDR45* band, ~508 bp), serving as a technical control for blood samples. **(B)** Sanger sequencing confirmation of *COL4A5* exon 23 skipping. The proband’s sequence demonstrates direct splicing of exon 22 to exon 24, with complete loss of the intervening exon 23 sequence (71 bp), confirming the presence of an aberrant transcript resulting from exon 23 skipping.

To compare the results of minigene testing and further investigate the impact of the *COL4A5* splice-site variant on endogenous mRNA processing, we performed RT-PCR on total RNA extracted from the proband’s skin tissue. The primers were designed to amplify a 537 bp fragment spanning exons 20–24 of *COL4A5*. As shown in Figure 4A, the proband’s sample (Lane 1) yielded a single amplified product shorter than both the expected wild-type *COL4A5* transcript (537 bp) and the internal control (*WDR45*, 508 bp), confirming *COL4A5* mRNA expression in skin tissue and indicating aberrant splicing. Sanger sequencing of this amplicon (Figure 4B) confirmed complete exon 23 skipping (71 bp), resulting in direct splicing of exon 22 to exon 24 and a transcript size of 466 bp (537–71 bp). Collectively, *in vivo* transcript analysis confirms that the variant (c.1587 + 4A > G) disrupts the splice donor site of intron 23, leading to exon 23 skipping and a transcript harboring a 71-bp in-frame deletion. This aberrant mRNA harbors a premature termination codon (p.S507Hfs*14) and is predicted to undergo nonsense mediated mRNA decay. This finding is consistent with *in vitro* minigene assay. *In vivo* and in vitro transcriptome analysis revealed that this variant produces an aberrant splicing pattern in tissues. Based on its cosegregation with the family’s phenotype and its effect on splicing, the variant was classified as “pathogenic” according to the 2015 ACMG/AMP guidelines and the 2023 ClinGen SVI splicing recommendations. The variant was absent from public population databases (PM2_Supporting), co-segregated with the disease phenotype in the family (PP1_Moderate), and is located at a splice donor site harboring multiple known pathogenic variants (PS1_Supporting). As specified in the ACMG framework, one very strong evidence plus additional moderate and supporting evidences is sufficient to reach a pathogenic classification.

## 4 Discussion

In this study, we identified a novel *COL4A5* splicing variant (c.1587 + 4A > G) in a Chinese family with XLAS via WES. This variant was confirmed by Sanger sequencing, highlighting the value of gene sequencing in etiological diagnosis of hereditary kidney diseases. Functional analyses—including minigene assay in cultured cells and *in vivo* RT-PCR of skin-derived RNA—provided supportive evidence for the variant’s role in abnormal splicing, and its segregation with disease in the family was consistent with a typical X-linked inheritance pattern.

Mutation type and locus are major determinants of phenotypic severity in XLAS, including age at kidney failure onset, susceptibility to SNHL, and risk of ocular abnormalities (5, 13). Therefore, the “Guidelines for the Diagnosis and Treatment of Alport Syndrome” (2) recommend that all individuals who may have Alport syndrome should undergo genetic testing to confirm the diagnosis and inheritance pattern and predict the age at onset of kidney failure. Assessing the pathogenicity of intronic variants poses a considerable challenge, as it largely relies on transcriptional analysis, which is frequently hampered by the low abundance of *COL4A5* transcripts in readily accessible cell types such as peripheral blood leukocytes. Minigene assays offer a practical alternative to assess splicing effects *in vitro*, although results must be interpreted cautiously given WT minigenes may occasionally produce abnormal splicing bands, risking false positives (6, 14). Moreover, splicing patterns can differ between tissues and between in vitro and *in vivo* conditions, emphasizing the need for in vivo validation. Given the difficulty of obtaining kidney specimens, skin tissue serves as a viable surrogate for transcript analysis, as normal epidermal basement membrane expresses *COL4A5* mRNA and protein (15, 16).

Splicing variants of *COL4A5* that generate premature termination codons are defined as truncating transcripts. Horinouchi et al. reported a median age at ESKD of 20 years in patients with truncating transcripts, compared with 29 years in those with non-truncating splicing mutations (6). In a cohort of 187 Chinese males with XLAS, Liu et al. documented median ages at ESKD of 22 and 39 years for truncating and non-truncating mutations, respectively (17). In our study, minigene assays demonstrated that the variant (c.1587 + 4A > G) results in aberrant splicing with skipping of exon 23, which disrupts the open reading frame and introduces a premature termination codon (p.S507Hfs*14). This truncated transcript is predicted to undergo nonsense-mediated mRNA decay. This splicing defect was confirmed by RT PCR and direct sequencing in skin fibroblasts, providing complementary *in vitro* and *in vivo* supportive evidence for the variant’s functional impact. In the present study, a 5-year-old male proband presented with microscopic hematuria. However, his brother and maternal uncle, who did not receive renin angiotensin aldosterone system inhibitors, progressed to kidney failure at 15 and 25 years of age, respectively. In contrast, the proband’s 39-year-old mother and 70-year-old maternal grandmother have exhibited only microscopic hematuria to date. Phenotypic heterogeneity within the family (e.g., mild microscopic hematuria in females vs. severe kidney failure in males) may reflect variable X-chromosome inactivation in females (18) and differences in gene penetrance influenced by genetic and environmental factors (3).

Previous studies in patients with XLAS harboring splice-site variants have established that positive α5(IV) chain expression in tissues can predict non-truncated transcripts (6). In this study, immunofluorescence staining revealed a complete absence of α5 chain expression in the proband’s skin basement membrane, suggesting that the patient may have XLAS associated with a truncated transcript. Subsequent skin reverse-transcription assay and mRNA sequencing of mutant cell model further verified our hypothesis. Despite being a less invasive and valuable approach for XLAS diagnosis, skin biopsy has limitations. Loss of α5(IV) staining in the skin basement membrane strongly supports XLAS, but a normal staining result does not fully exclude the disease, especially in some female carriers or individuals with certain missense and splice variants. Accordingly, skin immunofluorescence for α5 (IV) could represent a cost-effective surrogate for mRNA assays in predicting the prognosis of patients with XLAS. However, further large-sample studies are warranted to confirm this finding. Studies have found that approximately 12% of female patients with XLAS and 90% of male patients with XLAS develop hearing loss by the age of 40 years (1). Extrakidney manifestations (SNHL, ocular abnormalities) were absent in the young proband and affected females, consistent with the non-congenital nature of AS-related SNHL. In contrast, the proband’s brother and uncle had early-onset SNHL, potentially linked to the severely truncated transcript. Notably, none of the affected family members exhibited ocular abnormalities, a finding consistent with our previous study (19). This observation may be attributed to tissue-specific splicing patterns of *COL4A5* or ethnic disparities.

Elucidating alternative splicing patterns in XLAS is critical for prognosis assessment, genetic counseling, and the development of targeted therapies. Splicing modulation shows promise: Yamamura et al. demonstrated that exon-skipping strategies halted kidney failure progression in *COL4A5*-mutant mice, suggesting potential clinical applicability (20). Our study expands the *COL4A5* mutation spectrum and underscores the value of combining minigene assays with skin-derived transcript analysis for evaluating intronic splicing variants. However, gene splicing is highly tissue-specific, and our functional data may not reflect splicing patterns in the target organs (kidney, eye, inner ear), which have unique splicing regulatory mechanisms. Several limitations should be noted. Analysis based on a single family may limit extrapolation of the study results. Functional analyses were limited to transcript evaluation without protein assessment. We lack direct evidence of aberrant splicing in target organs and functional verification in tissue-specific models, limiting our ability to definitively link observed splicing to pathogenicity and restricting the generalizability of our findings. Future studies with larger cohorts, tissue specific splicing and protein function analyses are needed to verify the pathogenicity of this variant.

## 5 Conclusion

In summary, this study identified a novel intronic variant (c.1587 + 4A > G) in the *COL4A5* gene in a Chinese family with XLAS. Functional assays confirmed that this variant disrupts canonical splicing, most likely resulting in the skipping of exon 23. These findings expand the mutation spectrum of *COL4A5* associated with XLAS and enrich our understanding of the genotype–phenotype correlation in this disease Furthermore, our results confirm that transcript analysis is crucial for evaluating intronic variants, as it plays an essential role in the accurate assessment of variant pathogenicity.

## Data Availability

The data analyzed in this study is subject to the following licenses/restrictions: the datasets for this article are not publicly available due to concerns regarding participant/patient anonymity. Requests to access the datasets should be directed to the corresponding author. Requests to access these datasets should be directed to PQ qianpei2008@126.com.

## Footnotes

1
https://spliceailookup.broadinstitute.org/

2
https://rddc.tsinghua-gd.org/

3
http://www.fruitfly.org/seq_tools/splice.html

4
https://services.healthtech.dtu.dk/services/NetGene2-2.42/
